# Traumatic Dislocation of the Proximal Tibiofibular Joint: A Systematic Review and 10-year Experience From a Level 1 Trauma Center

**DOI:** 10.5435/JAAOSGlobal-D-21-00105

**Published:** 2022-05-03

**Authors:** Prashant V. Rajan, David E. Ramski, Nicholas M. Romeo

**Affiliations:** From the Department of Orthopaedic Surgery, Cleveland Clinic, Cleveland, OH (Dr. Rajan), and the Department of Orthopaedic Surgery, MetroHealth Medical Center, Cleveland, OH (Dr. Rajan, Dr. Ramski, and Dr. Romeo).

## Abstract

**Introduction:**

Traumatic proximal tibiofibular joint dislocations occur infrequently and are typically the result of high-energy trauma. These injuries can be a marker of limb injury severity because patients often sustain vascular injury and are at high risk of amputation. The purpose of this study was to present a systematic review of traumatic proximal tibiofibular joint dislocations and compare rates of associated injuries with a retrospective series of patients at a level 1 trauma center. The secondary objective was to report rates and clinical predictors of limb amputation.

**Methods:**

A systematic review was conducted, identifying three studies meeting eligibility criteria. A retrospective chart review was conducted identifying 17 skeletally mature patients with proximal tibiofibular dislocation treated from January 2010 to February 2021. A chart review extracted patient demographics, fracture patterns, open fracture, preoprative and postoperative peroneal nerve injury, vascular injury, and amputation. Binary logistic regression analysis was used to identify clinical predictors of outcomes.

**Results:**

Sixteen of 17 proximal tibiofibular injuries (94.1%) were associated with fracture, most commonly tibial shaft (n = 11, 68.75%). Twelve of 17 fractures (76.5%) were open. Five vascular injuries (29.4%) occurred requiring surgical intervention. Seven (41.2%) preoperative peroneal nerve deficits were noted; six had persistent deficits postoperatively or underwent amputation (average follow-up 31.3 ± 32.6 months). Two patients in the sample without preoperative peroneal nerve deficits were noted to exhibit them after fixation. Eight patients (47%) underwent an amputation, 7 (87.5%) of whom had an open fracture and 4 (50%) of whom had documented vascular injury.

**Discussion:**

Traumatic proximal tibiofibular fractures indicate severe injury to the lower extremity with high risk for nerve injury and possible amputation. Patients who present with vascular injury and open fracture in association with proximal tibiofibular joint disruption may be at elevated risk of amputation.

The proximal tibiofibular joint represents the synovial articulation between the proximal aspects of the posterolateral tibia and the anteromedial fibula.^1^ With high-energy injuries, traumatic dislocation of this joint can occur,^2^ although lower energy ligamentous strains and instability of this joint have also been recognized.^3,4,5,6,7,8^ There is overall a paucity of literature on traumatic dislocations of the proximal tibiofibular joint, although a few small case studies have been reported.^9,10,11,12,13,14,15,16,17,18,19,20,21^ There is similarly no systematic review of the literature on this topic.

A retrospective series by Herzog et al^2^ represents the largest collection of 30 patients with traumatic proximal tibiofibular dislocation, finding high rates of compartment syndrome, open fractures, and peroneal nerve palsy characteristic of high-energy injuries. The peroneal nerve, with close proximity to and tethered near the proximal tibiofibular joint,^22^ is at risk for injury. However, the series by Herzog et al did not specifically evaluate the preoperative and postoperative nerve injury rates.

The purposes of this study were (1) to conduct a systematic review of the literature to identify associated injuries of traumatic proximal tibiofibular joint dislocations, including rates of open fractures and vascular injury, (2) to perform a retrospective case series of patients undergoing treatment for traumatic proximal tibiofibular joint dislocation at a level 1 trauma center to compare rates of associated injuries with those identified in the literature, and (3) to assess for rates of nerve injury and recovery in association with proximal tibiofibular joint disruption and identify clinical predictors of need for amputation.

## Methods

**Figure 1:**
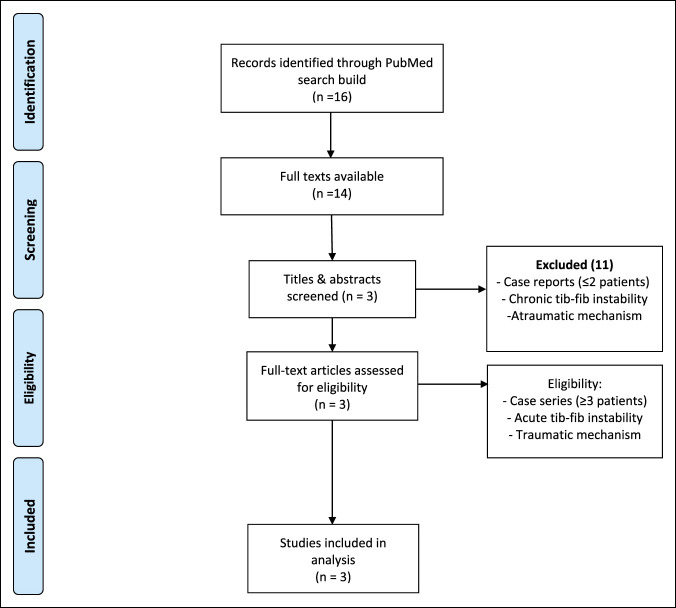
Flow diagram detailing the systematic review process used for the study.

A systematic review was conducted of MEDLINE to analyze studies of traumatic proximal tibiofibular joint dislocation (Figure 1). Specific search build was used to identify the terms “proximal tibiofibular” or “proximal tibio-fibular” and “trauma” in study titles. This resulted in a total of 14 studies available for a full-text review. Titles and abstracts were screened for eligibility criteria, which included studies involving ≥3 patients, subjects involving acute proximal tibiofibular joint dislocation (NOT chronic instability), and subjects sustaining such injuries by a high-energy, traumatic mechanism. This resulted in a total of three studies, which underwent full-text review to confirm eligibility and inclusion in the systematic review. These studies were then reviewed for rates of associated injuries, including open fracture, vascular injury, preoperative or postoperative nerve injury, and amputation (Table 1).

**Table 1:** Table of Studies Meeting Eligibility Criteria of Our Systematic Review of Traumatic Proximal Tibiofibular Joint Dislocation

Study	Study Sample	Sex (M/F)	Average Age	Average Follow-up	Open Fracture	Vascular Injury	Peroneal Nerve Deficit		Amputation
							Preoperative	Postoperative	
Gabrion et al (2004)^23^	8	8/0	31 yr	5.8 yr	8 (100%)	5 (62.5%)	5 (62.5%)	N/A	5 (62.5%)
Herzog et al (2015)^2^	30	26/4	40 yr	11 mo	19 (63%)	2 (6.7%)	10 (33%)	N/A	2 (6.7%)
Krukhaug et al (2019)^13^	3	2/1	44.3 yr	6 mo	1 (33%)	0	0	N/A	0

A retrospective chart review was then conducted of patients treated at a single level 1 trauma hospital for a 10-year period from January 1, 2010, to February 9, 2021. Patients were included in the study if they were older than 18 years and treated surgically for a proximal tibiofibular dissociation. Patients were excluded from the study if there was no documentation of preoperative and postoperative motor and sensory examination.

Patient age, sex, fracture pattern (Arbeitsgemeinschaft für Osteosynthesefragen [OA]/Orthopaedic Trauma Association [OTA] classification system), comorbidity status with American Society of Anesthesiology (ASA) classification, presence of open fracture, presence of vascular injury, preoperative and postoperative motor and sensory examination, and additional procedures conducted, including amputation and fasciotomy for compartment syndrome, were recorded. For continuous variables, averages were reported as means, SDs, and ranges. For categorical variables, averages were reported as percentages. Binary logistic regression analysis was used to identify clinical predictors of complications or outcomes, reported as adjusted odds ratios (ORs) and 95% confidence intervals (CIs). All data were analyzed with Minitab software (Minitab, LLC). Institutional review board approval was obtained before undertaking the study.

## Results

A total of 17 patients with 17 proximal tibiofibular dissociations were detected in the 10-year study period. Table 2 details the patient data and demographics for the study group. The average age of the group was 40.9 ± 12.6 years (range: 22 to 67), and 12 of the 17 patients were male (70.6%). The average follow-up was 28.6 ± 27.5 months (range: 6 to 99). All but two patients had the 1-year follow-up; the two remaining patients had 6- and 8- month follow-ups, and both went on to fracture union without the need for amputation. The most common mechanisms of injury were motorcycle (41.1%) and pedestrian struck (17.6%). Preoperative comorbidity ASA classifications were 7 type II (41.1%), 6 type III (35.3%), and 4 type IV (23.5%). As depicted in Table 1, 16 of the 17 proximal tibiofibular injuries (94.1%) were associated with a fracture: 11 tibial shaft fractures (68.75%), 3 proximal tibial/plateau fractures (18.75%), 1 tibial plafond fracture (6.25%), and 1 distal fibular fracture with syndesmotic injury (6.25%). Of the tibial shaft fractures, 6 (54.5%) were classified as OTA class C versus 5 OTA class B (45.5%).

**Table 2:** Patient Demographics and Data

Patient	Age (yr)	Sex	Fracture (OTA Classification)	Comorbidity (ASA Classification)	Mechanism	Open Fracture	Vascular Injury	Peroneal Nerve Deficit		Amputation^a^
								Preoperative	Postoperative	
1	42	M	42-C3	III	Motorcycle	+	+	+	N/A^b^	+
2	33	F	42-A3	II	Motorcycle	+	—	—	—	—
3	32	F	42-C3	II	Motorcycle	+	—	+	N/A	+
4	35	M	42-C3	II	Chainsaw	+	+	+	N/A	+
5	53	M	44-C3.1	III	Pedestrian struck	—	—	+	+	—
6	27	M	42-A3	IV	Motorcycle	+	+	+	N/A	+
7	48	M	42-C2	IV	Motorcycle	+	+	—	—	+
8	30	M	41-A2	II	Gunshot wound	—	—	—	—	—
9	60	M	41-A3	III	Motor vehicle	+	—	—	—	—
10	22	F	41-C3	IV	Bicycle	+	—	—	+	—
11	46	F	N/A	II	Motor vehicle	—	+	—	—	—
12	53	M	42-C3	II	Pedestrian struck	+	—	+	N/A	+
13	49	M	42-C2	III	Motorcycle	+	—		N/A	+
14	33	M	43-C3	IV	Fall from 10 feet	—	—		+	+
15	25	M	42-A3	III	Pedestrian struck	+	—	+	+	—
16	46	F	42-A3	II	Fall down stairs	+	—	—	—	—
17	67	M	42-A2	III	Motorcycle	—	—	—	—	—

a All amputations were below the knee (ie, transtibial).

b Postoperative nerve deficit was not applicable because patient received a limb amputation as definitive treatment of their injury.

Most of the fractures (12 of 17, 70.6%) were open. Five injuries (29.4%) were associated with a vascular injury requiring a repair or amputation. Seven injuries (41.2%) preoperatively were noted to have a peroneal nerve deficit. Six of these patients had persistent deficits postoperatively or proceeded to amputation at the final follow-up (average 13.5 ± 16.3 months) because of the severity of their injury. One patient who did not proceed to amputation had partial nerve function recovery but experienced severe pain consistent with complex regional pain syndrome. One of the seven patients did have full return of nerve function at 6- and 9-month follow-ups. There were two patients in the total sample without preoperative peroneal nerve deficits who were noted to exhibit them after their proximal tibiofibular fixation. No patient specifically presented with or developed compartment syndrome in the total sample.

Regarding surgical outcomes, a total of eight patients (47%) in the sample underwent an amputation, 7 (87.5%) of whom had an open fracture and 4 (50%) of whom had a vascular injury. Of these eight patients receiving an amputation, six patients (three with an open fracture alone and three with open fracture and vascular injury) had a partial amputation or a mangled extremity on presentation requiring amputation, one patient with an open fracture and vascular injury received a vascular repair with limb osteoplasty which failed requiring amputation 7 days later, and one patient with a pilon fracture without vascular injury but with residual peroneal nerve deficits required amputation 5 weeks later secondary to pain and dysfunction. The risk of amputation did not achieve statistical significance with open fracture (OR = 4.70 [95% CI, 0.12 to 177.52]) nor vascular injury (OR = 7.3062 [95% CI, 0.28 to 188.91]). Only one patient proceeded to nonunion of the proximal tibiofibular joint secondary to infection, requiring revision fixation. Six patients (35.3%) had their proximal tibiofibular implant removed secondary to loosening or discomfort.

## Discussion

**Figure 2:**
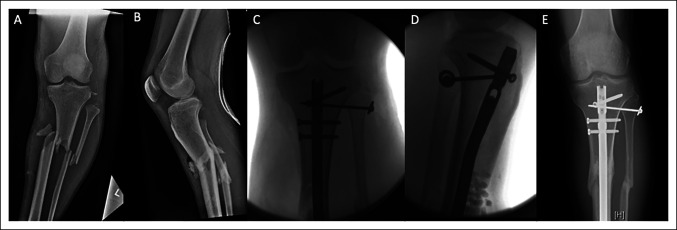
**A** and **B**, Original injury radiographs showing a Gustilo-Anderson grade IIIA open left tibia/fibula fracture (OTA classification 42-A3) with associated proximal tibiofibular dissociation. **C** and **D**, Intraoperative fluoroscopic images demonstrating excisional débridement of the anterior tibial wound with open reduction and intramedullary nail fixation of the tibial diaphyseal fracture with three proximal interlocking bolts. In addition, an open reduction and internal fixation of the proximal tibiofibular joint was done with 3.5 mm cortical screw and washer. **E**, Ten-month postoperative follow-up radiograph demonstrating union of the tibial and fibular diaphyseal fractures with maintained reduction of the proximal tibiofibular joint.

In the current series, patients sustaining a proximal tibiofibular injury had a high rate of open fracture (70.6%), vascular injury (29.4%), and preoperative peroneal nerve deficit (41.2%) characteristic of a high-energy, limb-threatening injury. In addition, the most common fracture morphology associated with proximal tibiofibular injury was a tibial shaft fracture (Figure 2) (68.75%), which is consistent with previous studies.^10,21^ Of the many case reports published on this topic,^9,10,11,12,13,14,15,16,17,18,19,20,21^ this systematic review was only able to identify three larger case series that have published the epidemiology of these injuries. Herzog et al^2^ published a similarly high rate of open fracture (76.7%) and peroneal nerve palsy (36%) with their series of 30 proximal tibiofibular injuries; however, their rate of vascular injury was lower at 6.7%. The smaller series of nine proximal tibiofibular injuries by Gabrion et al^23^ published a higher rate of vascular injury (56%), which more closely resembles the rate in our series.

The high rate of preoperative and postoperative peroneal nerve injury in these injuries stems from the close anatomic relationship of the peroneal nerve to the proximal tibiofibular joint, which has shown to be tethered approximately 2 cm distal to the fibular head.^22^ Our series, in similarity to that by Herzog et al,^2^ publishes a poor prognosis for these palsies because only one of seven patients recovered function on follow-up. Interestingly, two patients in our series sustained peroneal nerve palsies only after proximal tibiofibular joint fixation, suggesting possible iatrogenic intraoperative injury that did not recover. Although this can occur during the surgical approach or reduction, care must be taken to not place implants too distal or posterior on the fibular head, with cadaveric studies reporting a safe zone on the anterior 50% of the proximal 2 cm of the fibular head.^24^ This is the first study to differentiate preoperative and postoperative peroneal injury.

**Figure 3:**
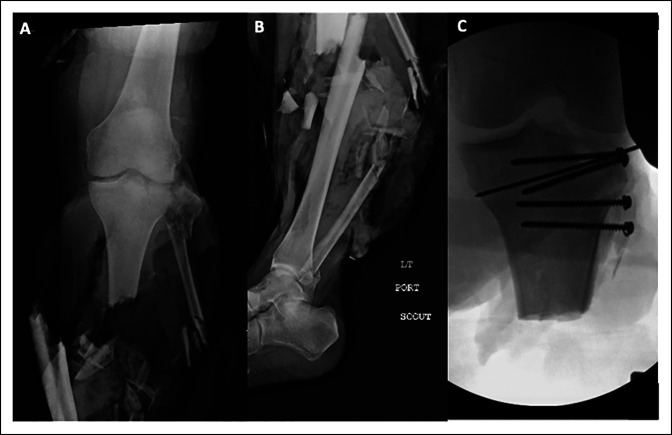
**A** and **B**, Original injury radiographs depicting Gustilo-Anderson grade IIIC open tibia and fibula diaphyseal fractures with severe soft-tissue injury and partial limb amputation (OTA classification 42-C3). There is a proximal fibular fracture with proximal tibiofibular joint dissociation and a simple split fracture of the lateral tibial plateau articular surface. **C**, Intraoperative fluoroscopic radiographs demonstrating revision amputation of the injured extremity with open reduction and internal screw fixation of the lateral tibial plateau split fracture in addition to open reduction and internal fixation of the proximal tibiofibular joint with 3.5 mm cortical screws.

In line with the high-energy mechanisms of proximal tibiofibular dislocation, our series uniquely reports a high rate of limb amputation (Figure 3) (47%). This is in contrast to the larger series published by Herzog et al, in which only two of 30 patients (6.5%) required an amputation secondary to severe soft-tissue damage. Gabrion et al^23^ published a higher rate of amputation at 33%. Although this study was unable to find statistically significant clinical predictors of amputation, it is expected that a percentage of these injuries within the above range will sustain unsalvageable soft-tissue damage requiring amputation. A larger, prospective series would provide more clarification on this statistic.

The current series also uniquely reports a high rate of implant removal (35.3%) after proximal tibiofibular fixation and subsequent union. All of these patients had their implants removed because of discomfort or loosening. Studies have demonstrated alterations in ipsilateral ankle biomechanics and clinical dysfunction after proximal tibiofibular arthrodesis,^25-27^ indicating the dynamic nature of this complex joint. Although the existing landscape of literature is focused on different fixation strategies for chronic sports-related proximal tibiofibular instability,^3,4,5,6,7,8^ there is a role for these investigations in the acute traumatic dislocation.

There are several limitations to the study. This was a retrospective study that is limited by the information reported in the chart review and by the inherent biases of a retrospective data set. The series has a small overall sample size for analysis and is likely underreporting the number of proximal tibiofibular injuries sustained, given that these rare injuries are identified by their subsequent fixation. In addition, there is likely a low representation of commonly associated injuries, such as compartment syndrome. However, this study represents one of the largest series of patients sustaining traumatic proximal tibiofibular dislocations and identifies them as high-energy injuries associated with open tibial shaft fractures, peroneal nerve palsies, vascular injury, and amputation.
